# Global and Geographically Weighted Quantile Regression for Modeling the Incident Rate of Children’s Lead Poisoning in Syracuse, NY, USA

**DOI:** 10.3390/ijerph15102300

**Published:** 2018-10-19

**Authors:** Zhen Zhen, Qianqian Cao, Liyang Shao, Lianjun Zhang

**Affiliations:** 1 Department of Forest Management, School of Forestry, Northeast Forestry University, Harbin 150040, Heilongjiang, China; zhzhen@syr.edu; 2 Department of Forest and Natural Resources Management, State University of New York College of Environmental Science and Forestry, One Forestry Drive, Syracuse, New York, NY 13210, USA; qcao03@syr.edu (Q.C.); shaoliyang@gmail.com; (L.S.)

**Keywords:** incidence rate of children’s blood lead poisoning, elevated blood lead level, quantile regression, geographically weighted quantile regression

## Abstract

*Objective*: The purpose of this study was to explore the full distribution of children’s lead poisoning and identify “high risk” locations or areas in the neighborhood of the inner city of Syracuse (NY, USA), using quantile regression models. *Methods*: Global quantile regression (QR) and geographically weighted quantile regression (GWQR) were applied to model the relationships between children’s lead poisoning and three environmental factors at different quantiles (25th, 50th, 75th, and 90th). The response variable was the incident rate of children’s blood lead level ≥ 5 µg/dL in each census block, and the three predictor variables included building year, town taxable values, and soil lead concentration. *Results*: At each quantile, the regression coefficients of both global QR and GWQR models were (1) negative for both building year and town taxable values, indicating that the incident rate of children lead poisoning reduced with newer buildings and/or higher taxable values of the houses; and (2) positive for the soil lead concentration, implying that higher soil lead concentration around the house may cause higher risks of children’s lead poisoning. Further, these negative or positive relationships between children’s lead poisoning and three environmental factors became stronger for larger quantiles (i.e., higher risks). *Conclusions*: The GWQR models enabled us to explore the full distribution of children’s lead poisoning and identify “high risk” locations or areas in the neighborhood of the inner city of Syracuse, which would provide useful information to assist the government agencies to make better decisions on where and what the lead hazard treatment should focus on.

## 1. Introduction

Lead is an important toxic mental that induces numerous adverse clinical outcomes and antisocial behavior in children and adults [1,2]. Lead exposure plays a significant role in life course since prenatal lead exposure could bring forth lasting damage to childhood, adolescence, earlier in adult life or across generations [1]. Both prenatal and postnatal exposure to lead had relationship with reported antisocial acts [2]. A number of studies have shown that even low lead exposure in early childhood will cause behavior, hearing, and learning problems in children’s growth and development. No lead level is considered safe [3,4,5,6,7]. It is known that the major environmental lead sources include lead-based paint, lead in indoor dust, lead in soil, and lead in water [5,8,9]. Children under six years of age have the highest exposure to lead due to greater hand dust contamination, frequent hand-to-mouth transfer, and higher absorption rates [10]. The most common physiological effects of lead poisoning include neurological and neurobehavioral, lower IQ, and slowed growth and anemia [1].

During the last decades the U.S. federal and state governments have devoted considerable efforts for lead hazard screening, mitigation, and control via intervention regulations and education programs. For example, the Lead Hazard Control Program of New York have assisted in reducing lead paint hazards in houses and provided lead inspection for rental properties and homes built before 1978 in the city of Syracuse (NY, USA), [11]. Although the national surveys and local surveillance data indicate that children’s blood lead level (BLL) (e.g., ≥10 g/dL) has declined over time [12,13,14,15], the incident of lead poisoning remains relatively high in some inner cities of USA. For example, in the city of Syracuse, a total of 4903 children under the age of 6 years were tested at least once in 2011. The screening results indicated that 168 (3.43%) of these children had BLL ≥10 μg/dL and 900 (18.36%) had BLL ≥ 5 μg/dL [16,17].

Therefore, lead poisoning is still a concern of public health in the inner and old industrial cities. The urban environment usually suffers from historically emitted leaded gasoline, smelters, iron and steel production, lead-acid-battery manufacturing, nonferrous (brass and bronze) foundries, landfills, waste incinerators, sewage sludge incinerators, hazardous waste sites, power plants (refineries/coal burning), and older homes that contain lead based paint/pipes [18]. Studies have shown that the incident rate of children’s lead poisoning was associated with environmental lead hazard exposure and socio-economic status such as the population density, proportion of houses built before 1950, percentage of poverty level and minority [19,20]. For example, Haley and Talbot [21] and Morrison et al. [22] showed that the age of houses was one of the most significant factors for predicting the children’s blood lead levels. However, in reality, the relationships between children’s blood lead levels and environment risk factors are not simple and homogeneous in nature, because the socio-economic status of households may be clustered in geographical areas of the inner cities [20,23]. The governments’ lead hazard control projects have also changed the geographical distributions of lead contamination exposure over time. All those factors make the relationships between children’s lead exposure/poisoning and environmental risk factors complicated and heterogeneous over space [19,24].

Regression models have been widely used in statistical and ecological analyses of epidemiological research. However, most published regression models are global in nature, assuming that the relationships between response variable and risk factors are constant or spatially homogenous. This assumption may not be reasonable for many cases in health studies [25]. From a policy-making viewpoint, a global model is useful to summarize how health outcomes are shaped, but does not provide detailed information for effective and efficient regulations and interventions [26]. On the other hand, the spatially varying relationships may provide us opportunities to search and identify the key and specific areas across a large geographical study region. Particularly, a localized regression method, geographically weighted regression (GWR), has become popular in different disciplines in recent years (e.g., [11,27,28,29,30,31,32,33]). The applications of GWR in epidemiological research helped to identify the “hot spots” of the inner cities where the disease rate is more severe than other areas, which may be more important and useful to policy makers for the purpose of planning and management [24,34].

In addition, most regression models, including both global and GWR models, focus on the “average” relationships between response variable and predictor variables, and provide the prediction on the conditional mean (i.e., central behavior) of the response variable given the values of the predictor variables [35]. In practice, people may also be interested in the conditional percentiles (or quantiles) and/or extremes of a response variable distribution in a linear model in order to explore more complete and comprehensive relationships between response variable and predictor variables [36,37]. For this purpose, quantile regression has been used in different study fields since its development [38]. Zhang et al. compared quantile regression with other regression methods for modeling the self-thinning upper boundary line of forest stands [39]. Koenker and Hallock used quantile regression to study re-employment bonus at different quantiles in microeconomics [40]. Levin applied quantile regression in education to study the class size and peer effects [41]. Wei et al. utilized quantile regression for growth charts where the percentile curves are commonly used to screen abnormal growth [42]. The main advantages of quantile regression include: (1) it is flexible to model data with heterogeneous conditional distributions and robust to outliers; (2) it makes no distributional assumption about the error term in the model so that it provides efficient estimates of model coefficients under non-Gaussian conditions; and (3) it can estimate multiple rates of change (slopes) from minimum to maximum of the response variable, providing a more complete view of the relationships between variables. Thus, quantile regression is especially useful in applications where limiting factors or extremes are more important than the averages, e.g., the upper quantiles of air pollution levels are more critical to public healthcare [36,37,43].

Further, Chen et al. developed geographically weighted quantile regression (GWQR) to integrate the GWR methodology with the quantile regression framework [44]. This innovative approach provides a foundation for modeling spatially nonstationary relationships between variables at a range of conditional quantiles of the response variable distributions. Since then, however, to our best knowledge there were very limited applications of GWQR. The objective of this study was to apply global quantile regression (QR) and geographical weighted quantile regression (GWQR) to model the relationships between children’s lead poisoning and environmental risk factors referenced at different quantiles, rather than at the conditional mean, of the response variable. Hopefully, quantile regression models enabled us to explore the full distribution of children’s lead poisoning and identify “high risk” locations or areas in the neighborhood of the inner city of Syracuse, NY, USA.

## 2. Theoretical Background

We briefly describe the quantile regression techniques used in this study.

### 2.1. Quantile Regression 

For a random response variable Y with a cumulative distribution function (***cdf***) F(y)=Pr(Y≤y), the ***τ***th quantile of Y is defined as the inverse of the ***cdf*** at τ, that is the value of Y such that F(Y)=Pr(Y≤ξ)=τ, where 0 < τ < 1. Thus, the proportion of the population with the response variable below ξ(τ) is τ. For example, the 90th quantile of the standard normal distribution is ξ(τ=0.9) = 1.284 (i.e., the Z-score value). The inverse function $Q\left(\tau \right)=F^{-1}\left(\tau \right)=\inf\left(y:F\left(Y\right)\geq \tau \right)$ is called the quantile function of F(Y). The general τ-th sample quantile ξ(τ), which is the analogue of Q(τ), can be obtained by minimizing:

(1) $$\xi \left(\tau \right)=\min\overset{n}{\underset{i=1}{\sum }}\rho_{\tau }\left(Y_{i}-\xi \left(\tau \right)\right)=\min\left[\left(1-\tau \right)\underset{Y_{i}<\xi }{\sum }\left(Y_{i}-\xi \left(\tau \right)\right)+\tau \underset{Y_{i}\geq \xi }{\sum }\left(Y_{i}-\xi \left(\tau \right)\right)\right]$$

where the loss function $\rho_{\tau }$ assigns a weight of ***τ*** to positive residuals $Y_{i}-\xi \left(\tau \right)$ and a weight of (1 − τ) to negative residuals [45].

Quantile regression (QR) is designed to model the effects of p predictor variables X on the conditional percentiles or quantiles of a response variable, such that Q(τ|X)=Xβ(τ)+ε(τ), where 0 < τ < 1. However, the model error term **ε**(τ) is unspecified and is only assumed that **ε**(τ) satisfies the quantile restriction Q(ε(τ)|X)=0 [38]. The quantile regression coefficients can be obtained by solving for any quantile 0 < τ < 1:

(2) $$\hat{\beta }\left(\tau \right)=\min\overset{n}{\underset{i=1}{\sum }}\rho_{\tau }\left(Y-X\beta \left(\tau \right)\right)$$

where $\rho_{\tau }$ is a V-shaped piecewise loss function. For the case of τ = 0.5, the quantile regression is the median regression, also known as L_1_ regression estimator.

### 2.2. Geographically Weighted Quantile Regression (GWQR)

To investigate the spatial heterogeneity of a regression relationship, the data must be collected with the location coordinates (u_i_, v_i_) for each observation i (i = 1, 2,…, n). The local information leads to estimate the localized regression coefficients of the relationship of interest. Geographically weighted quantile regression (GWQR) developed by [44] is expressed as follows:

(3) $$Y_{i}\left(\tau \right)=X_{i}\beta \left(\tau \right)\left(u_{i},v_{i}\right)+\varepsilon_{i}\left(\tau \right)=\beta_{0}\left(\tau \right)\left(u_{i},v_{i}\right)+\overset{p}{\underset{k=1}{\sum }}\beta_{k}\left(\tau \right)\left(u_{i},v_{i}\right)X_{\mathrm{ki}}+\varepsilon_{i}\left(\tau \right)$$

where Y_i_ is the response variable, X_k_ is a set of p predictor variables (k = 1, 2, …, p), ε_i_ is the error term of the conditional τ-th quantile function, and $\beta_{0}\left(\tau \right)\left(u_{i},v_{i}\right),\beta_{1}\left(\tau \right)\left(u_{i},v_{i}\right),\ldots ,\beta_{p}\left(\tau \right)\left(u_{i},v_{i}\right)$ are the local quantile regression coefficients for the τ-th quantile at the i-th location. The model coefficient $\beta_{k}\left(\tau \right)\left(u_{i},v_{i}\right)$ measures the change in a specified quantile τ of the response variable corresponding to one unit change in the predictor variable. The comparison between percentiles or quantiles of the response variable may reveal where a specific percentile or quantile is more affected by the set of predictor variables than others.

The estimation procedure of GWQR is as follows: (i) for one particular location i (the center) draw a circle of a given radius defined by a kernel function, (ii) compute a weight for each neighboring observation j according to the distance d_ij_ between the location j and center i, and (iii) estimate the model coefficients using linear programming optimization [44]. Thus, for a given regression point (u_0_, v_0_), the solution of the GWQR coefficients for the τ-th quantile in Equation (3) can be obtained by minimizing the geographically weighted loss function using the data within the kernel window:

(4) $$\overset{n}{\underset{i=1}{\sum }}\rho_{\tau }\left(Y_{i}\left(\tau \right)-\beta_{0}\left(\tau \right)\left(u_{0},v_{0}\right)-\overset{p}{\underset{k=1}{\sum }}X_{\mathrm{ik}}\beta_{k}\left(\tau \right)\left(u_{0},v_{0}\right)\right)\cdot W_{0}$$

where W_0_ is the spatial weights defined by a kernel function K (d_0i_/h) where h is the bandwidth and d_0i_ is distance between each neighboring location i and the regression point (u_0_, v_0_). Note: there is no explicit form available for the solution of the model coefficient vector in Equation (4). Instead, it can be equivalently formulated as a linear programming optimization problem [45,46].

Letting $\left(u_{0},v_{0}\right)=\left(u_{i},v_{i}\right)$ (i = 1, 2,…, n), the estimator ${\hat{\beta }}_{k}\left(\tau \right)\left(u_{i},v_{i}\right)$ (k = 0, 1, 2, …, p) for each GWQR coefficient can be obtained. Thus, the corresponding estimated τ-th quantile is calculated by

(5) $${\hat{Q}}_{\tau }\left(X_{i},u_{i},v_{i}\right)=X_{i}\hat{\beta }\left(\tau \right)\left(u_{i},v_{i}\right)={\hat{\beta }}_{0}\left(\tau \right)\left(u_{i},v_{i}\right)+\overset{p}{\underset{k=1}{\sum }}X_{\mathrm{ik}}{\hat{\beta }}_{k}\left(\tau \right)\left(u_{i},v_{i}\right)$$

where $\hat{\beta }\left(\tau \right)\left(u_{i},v_{i}\right)$ is the vector of regression coefficient estimates and X_i_ denotes the vector of observed predictor variables at the i-th location $\left(u_{i},v_{i}\right)$. More details on the GWQR coefficient estimates, standard errors of regression coefficients, kernel function and bandwidth selection, and the assessment of spatial non-stationarity can be viewed in [44].

## 3. Materials and Methods

### 3.1. Data

The surveillance data of children’s blood lead level (BLL) in the city of Syracuse, NY, USA were provided by the Onondaga County Health Department (OCHD). All lead testing results were reported to the New York State Department of Health via what is currently called “LeadWeb”. The dataset included the children’s lead screening tests from 2007 to 2011 in the city of Syracuse. To avoid the bias caused by the follow-ups, we chose only the first test of each child in the surveillance dataset. The lead test records included children’s birthday, test date, test type, gender, race and primary home address. For the purpose of ethical/human subject protection, the data that we received were modified for confidentiality by the following measures: (1) the names of the subjects were masked and (2) the locations of the homes were de-identified by the averaged values of latitude and longitude for each census block. There were a total of 24,222 observations. All the records were geo-coded by the reference data of 2010 TIGER/Line Shapefiles provided by U.S. Census Bureau (i.e., the geographical partition unit is the census block). By merging the blood lead test database with the census block map layer in ArcGIS 10 (Environmental Systems Research Institute, RedLands, CA, USA), a total of 1393 census blocks with children lead screening records were found, out of the 2350 total number of census blocks in the city of Syracuse [16].

In 1991, the Center for Disease Control and Prevention (CDC) defined BLLs ≥10 µg/dL as the “level of concern” for children aged 1–5 years. In 2012, CDC reduced the children’s BLL threshold of concern from 10 micrograms per deciliter (μg/dL) to 5 μg/dL (a.k.a. reference or elevated BLL) in order to fully address the extent of the childhood lead poisoning in USA [47]. In this study, we counted the percentage or incident rate of children whose BLL ≥ 5 µg/dL in each census block. If there was no child having BLL ≥ 5 µg/dL, we recorded 0 for the census block. Otherwise, the ratio of the number of children with BLL ≥ 5 µg/dL to the total number of tested children was computed and recorded for the census block, which produced the response variable Y between 0 and 1.

We chose three predictor variables based on previous studies [16]. The building year and town taxable values (in thousand dollars) of the residential houses in a census block were averaged in ArcGIS 10 to get the value at the census block level. The soil lead concentration (ppm) was based on [48], which collected and analyzed 3000 soil samples across the city of Syracuse in the summer of 2003 and 2004. Because the town taxable values were highly skewed, natural logarithmic transformation was used on this predictor variable.

**Figure 1:**
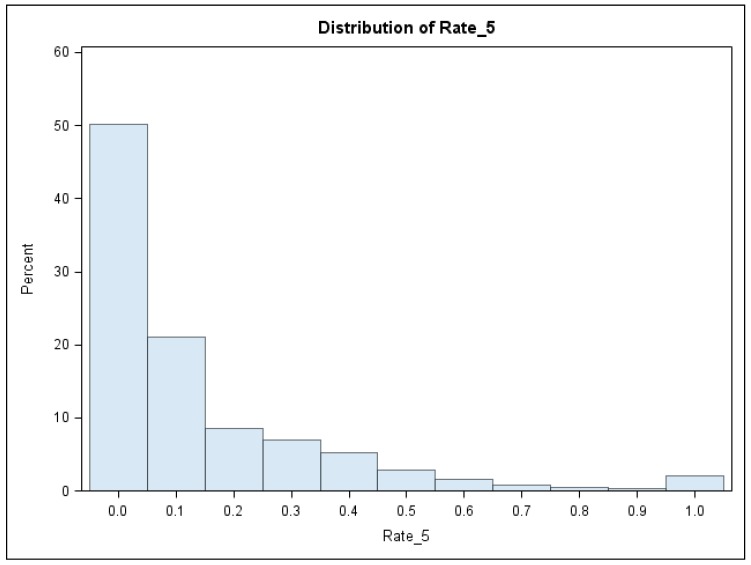
The histogram of the observed incident rate of children’s BLL ≥ 5 µg/dL in Syracuse, NY, USA.

In summary, the response variable was the incident rate (Y) of children’s BLL ≥ 5 µg/dL in each census block, and the three predictor variables were (1) building year (X_1_), (2) natural logarithm of town taxable values (in thousand dollars) (X_2_), and (3) soil lead concentration (ppm) (X_3_). The descriptive statistics of the response variable and three predictor variables were listed in Table 1. The histogram of the response variable is illustrated in Figure 1. The distribution of the response variable was summarized and showed in Table 2. For example, the 50th quantile (median) was 0.04, the 75th quantile 0.18, and the 90th quantile 0.40. Thus, ξ(τ=0.75) = 0.18 indicated that 75% of these 1393 census blocks had less than 18% children’s BLL ≥ 5 µg/dL among all tested children in the census block, while 25% of these 1393 census blocks (or 348 blocks) had 18% or more children’s BLL ≥ 5 µg/dL among all tested children in the census block. Those (348) census blocks may be considered “high risk” areas of children lead poisoning because about 20% of children’s BLL were over the elevated BLL, which should be identified for lead control treatment.

In general, the soil lead concentration was negatively associated with the building year (Pearson correlation coefficient = −0.54), meaning that the older the house, the higher the lead concentration in the soil. The Pearson correlation between the house building year and its town taxable value was +0.40, indicating that older houses had lower taxable values. The Pearson correlation coefficient between the town taxable values and soil lead concentration was −0.38. Given the relatively lower correlations between the three predictor variables, there was no severe multicollinearity problem in the modeling and prediction processes [49].

### 3.2. Methods

In this study, global quantile regression (QR) was firstly applied to model the incident rate of children’s BLL ≥ 5 µg/dL. Then, the geographically weighted quantile regression (GWQR) was utilized to explore the spatial heterogeneity of the response variable across the geographical areas of the inner city of Syracuse.

#### 3.2.1. Regression Model

We chose the following linear models for both global and GWR quantile regression to explore the relationship between the incident rate (Y_i_) of children’s BLL ≥ 5 µg/dL and three environment risk factors at the four quantiles of Y_i_ (τ = 0.25, 0.50, 0.75, and 0.90), respectively:

(6) $$Y_{i}\left(\tau \right)=\beta_{0}+\beta_{1}X_{1i}+\beta_{2}X_{2i}+\beta_{3}X_{3i}+\varepsilon_{i}$$

(7) $$Y_{i}\left(\tau \right)=\beta_{0}\left(u_{i},v_{i}\right)+\beta_{1}\left(u_{i},v_{i}\right)X_{1i}+\beta_{2}\left(u_{i},v_{i}\right)X_{2i}+\beta_{3}\left(u_{i},v_{i}\right)X_{3i}+\varepsilon_{i}$$

The SAS procedure PROC QUANTREG was used to fit the global quantile regression models (Equation (6)) [50], and the SAS macro provided by [51] was used to fit the GWQR models (Equation (7)).

#### 3.2.2. Bandwidth Selection for GWQR

An optimal bandwidth may be selected in terms of some criterion. There are three common ways of choosing the bandwidth: (1) subjective choice, (2) based on the smallest cross-validation error, and (3) based on the smallest Akaike Information Criterion (AIC) [29,39]. In this study, we used AIC to decide the optimal bandwidth and related kernel function for estimating each regression coefficient for each geographic location i and each predictor variable.

#### 3.2.3. Assessment of Spatial Nonstationary

To evaluate the spatial variation in regression coefficients of GWQR, we followed the approach in [44]. At a specified quantile, the interquartile range (IQR) of the local coefficient estimates computed by GWQR was compared with the standard error of the global estimates derived with a traditional QR. When IQR is twice as large as the standard error, it indicates that spatial non-stationarity exists in the relationships between response variable and its accompanying predictor variables.

## 4. Results

**Figure 2:**
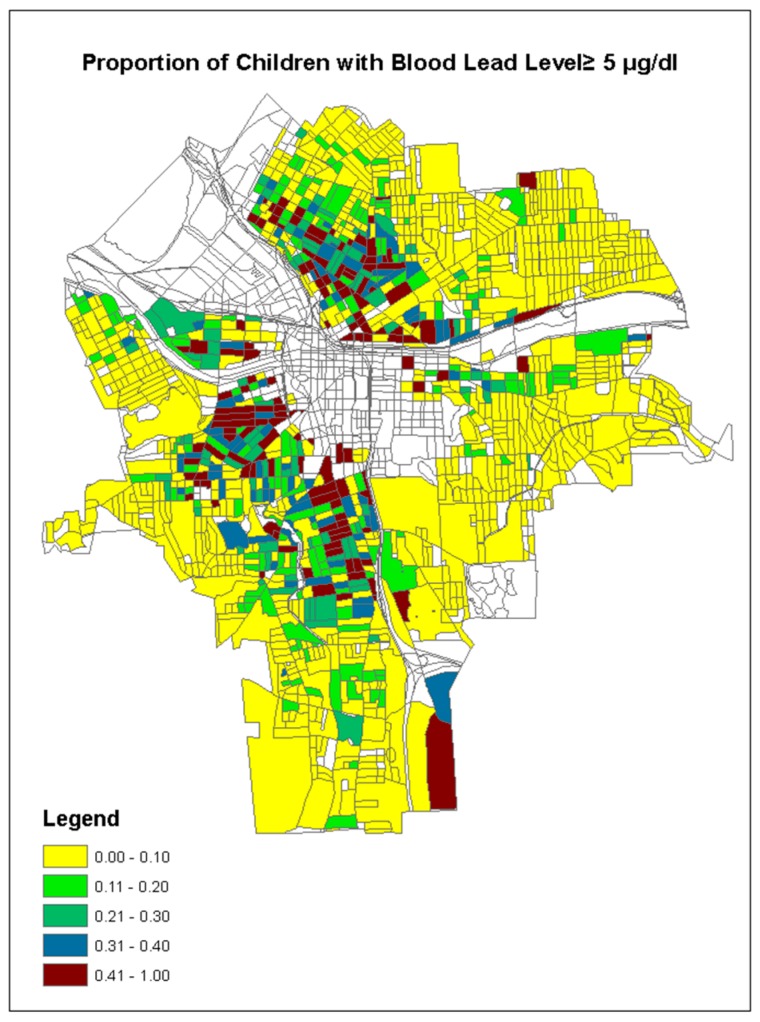
Geographical map of the observed incident rate of children’s BLL ≥ 5 µg/dL in the city of Syracuse, NY, USA, (the blanks are non-residential areas).

**Figure 3:**
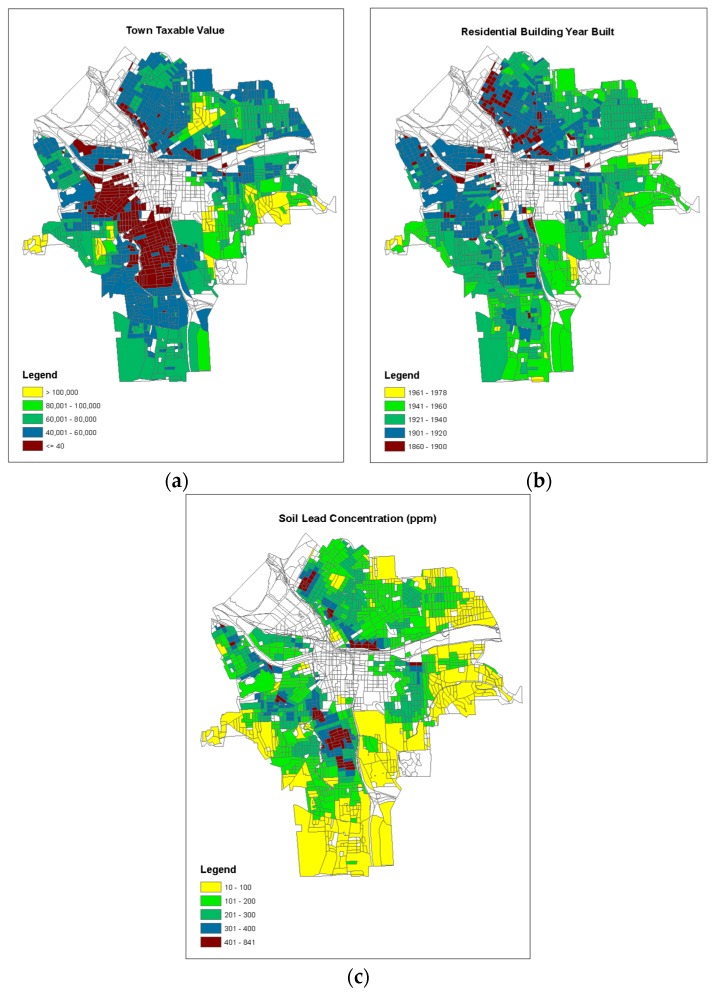
Geographical map of (**a**) the building year of residence; (**b**) the town taxable value of residence; and (**c**) the soil lead concentration in the city of Syracuse, NY, USA, (the blanks are non-residential areas).

The geographical map of the observed proportion or incident rate of children’s BLL ≥ 5 µg/dL is illustrated in Figure 2, and the geographical maps of the three predictor variables are shown in Figure 3. The higher incident rates of children lead poisoning existed in the center of the city, along the northern and southern sides of the non-residential area (commercial centers), where the residential houses were relatively older (built before 1920) with lower taxable values (<40 K, especially on the southern side), as well as relatively higher soil lead concentration (Figure 3).

**Figure 4:**
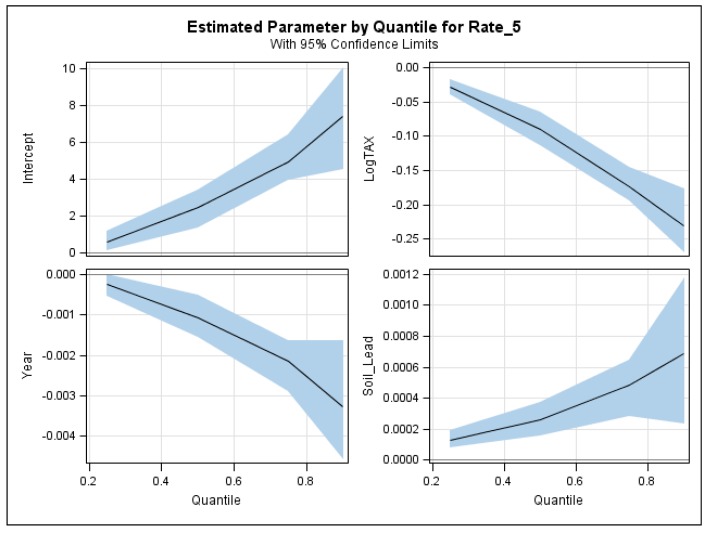
The model coefficient estimates of the global quantile regression models at different quantiles.

Global quantile regression models were fitted with the three predictor variables at τ = 0.25, 0.50, 0.75 and 0.90 quantiles (Table 3). It was evident that at each quantile, the regression coefficients of both building year and town taxable values were negative, indicating that the incident rate of children lead poisoning reduced with newer buildings and/or higher taxable values of the houses. In contrast, soil lead concentration was positively related to the incident rate of children lead poisoning, implying that higher soil lead concentration around the house may cause higher risks of children’s lead poisoning. Further, these negative or positive relationships between response variable and three environmental factors became stronger for larger quantiles (Figure 4).

In terms of statistical testing on the regression coefficients, the predictor variables taxable values and soil lead were statistically significant at all four selected quantiles, while the building year was significantly related to the incident rate of the children’s lead poisoning at the 0.50, 0.75 and 0.90 quantiles, but not important at the 0.25 quantile (Table 3). In order to compare the importance of the three predictor variables, all variables in Equation (6) were standardized to mean = 0 and standard deviation = 1 and the four global quantile regression models were re-fitted. The standardized model coefficients (STB) were also listed in Table 3. It was interesting that the town taxable value was the most important predictor variable at each quantile, followed by the soil lead concentration around the house which had similar level of (positive) impact as the taxable value. In contrast, the building year had much smaller (negative) impact on the incident rate of children lead poisoning than the taxable value of the house (Table 3).

We found that the existence of spatial autocorrelation and heterogeneity in the observed incident rate of children’s lead poisoning and the model residuals of the four global quantile regression models (Table 4). The positive Moran’s I values indicated that the “high risk” census blocks were neighboring with the “high risk” blocks, while the “low risk” census blocks were neighboring with the “low risk” blocks across the inner city of Syracuse, NY, USA. Although the values of the Moran’s I were not particularly large, these spatial autocorrelations were statistically significant. In addition, the Moran’s I became smaller for in the model residuals for larger quantiles, implying that “high risk” census blocks (i.e., more children with BLL ≥ 5 µg/dL) were less spatially clustered (Table 4).

The summary statistics of GWQR coefficient estimates were listed for the four selected quantiles in Table 5, including the mean, median, minimum and maximum of these 1393 sets of GWQR model coefficients. It was clear, in terms of the means and medians of these spatially varying model coefficients, the patterns and trends of the coefficients were similar to those of the global QR model coefficients: (1) the coefficients were negative for the building year and taxable values, but positive for the soil lead concentration; and (2) these negative or positive relationships became stronger for larger quantiles (Table 5).

However, across the geographical study area, the GWQR model coefficients varied from location to location. One way of assessing the spatial heterogeneity of the coefficients is to use the interquartile ranges (IQR) of the localized coefficients compared with the standards error of the global model coefficients. If the IQR is twice as large as the standard error, it indicates that spatial non-stationarity exists in the relationships between response variable and accompanying predictor variables [44]. Table 5 indicated that most IQRs of the localized model coefficients were at least twice the standard errors of the global model coefficients, except for soil lead concentration at τ = 0.25 and 0.90. Our results suggested that the relationships between the incident rate of children lead poisoning and environmental risk factors indeed varied across the inner city of Syracuse.

**Figure 5:**
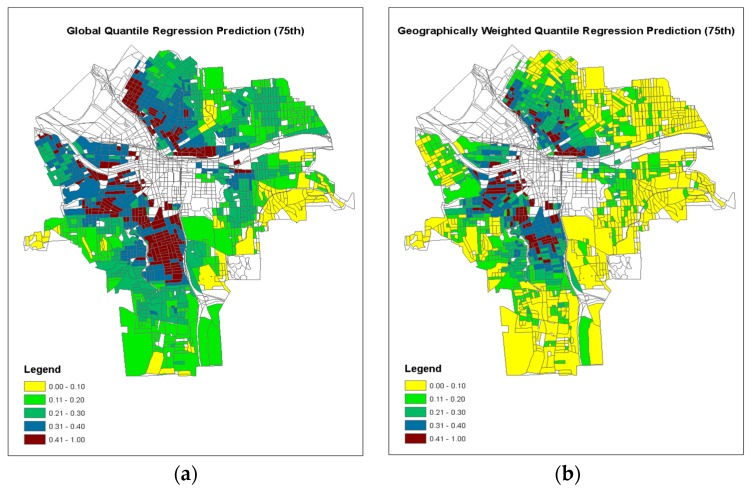
Model predictions from (**a**) global QR model at τ = 0.75; and (**b**) GWQR model at τ = 0.75.

Figure 5 illustrates the model predictions from both global QR model and GWQR model at the quantile τ = 0.75, because the observed 75th quantile of the response variable was 0.18 (Table 2), indicating that 25% of these 1393 census blocks (or 348 blocks) had 18% or more children’s BLL ≥ 5 µg/dL among all tested children in the census block. Those census blocks may be considered “high risk” areas of children lead poisoning and should be identified for lead control treatment. The predictions from the global QR (τ = 0.75) model show relatively larger areas of the inner city of Syracuse would have 20% or more children having elevated BLL ≥ 5 µg/dL in those census blocks, or even >40% of children having BLL ≥ 5 µg/dL in those census blocks (Figure 5a). One the other hand, the predictions from the GWQR (τ = 0.75) model show much smaller areas, or more specific “spatial spots” of 20% or more children having elevated BLL ≥ 5 µg/dL in those census blocks (Figure 5b), which may provide more useful information to the government agencies for the lead hazard control programs.

**Figure 6:**
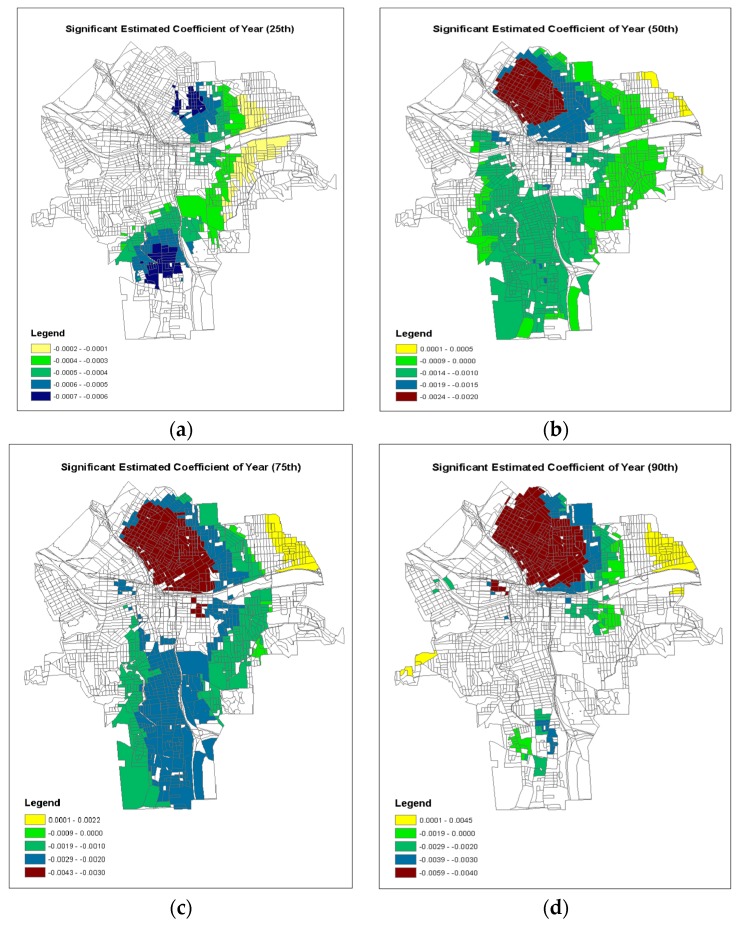
Geographical map of the significant coefficients (±1.96) of GWQR for Building Year: (**a**) 25th; (**b**) 50th; (**c**) 75th; (**d**) 90th.

**Figure 7:**
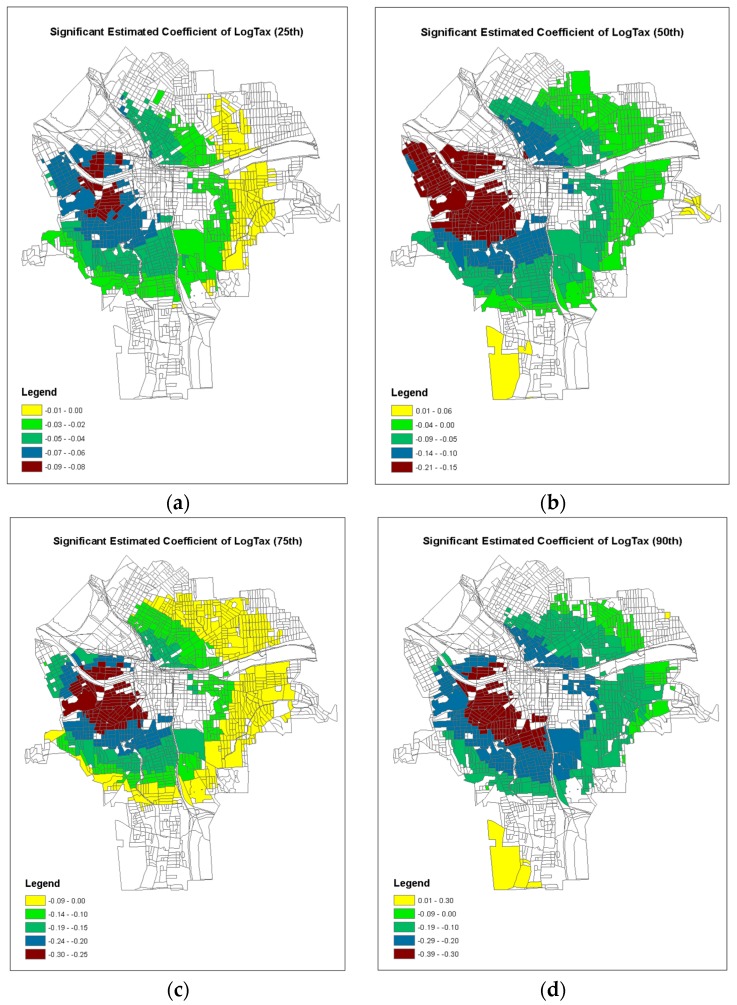
Geographical map of the significant coefficients (±1.96) of GWQR for LogTax: (**a**) 25th; (**b**) 50th; (**c**) 75th; (**d**) 90th.

**Figure 8:**
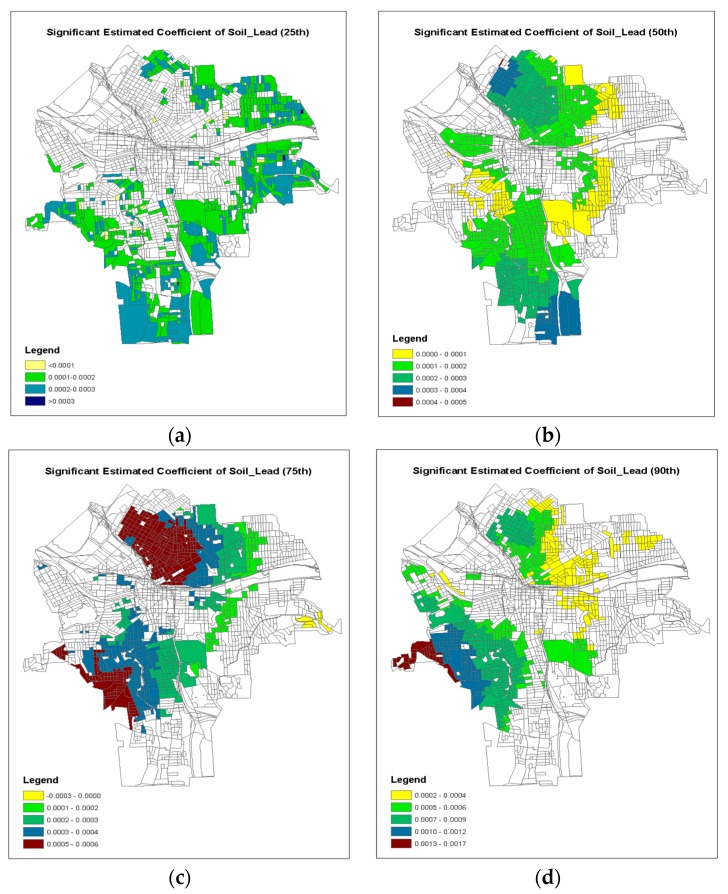
Geographical map of the significant coefficients (±1.96) of GWQR for Soil_Lead: (**a**) 25th; (**b**) 50th; (**c**) 75th; (**d**) 90th.

In addition, the GWQR modeling method produced the model coefficients for each location in the study area at any specific quantile. Those sets of model coefficients can be mappable using geographic information system like ArcGIS. Following [44], we constructed the geographic maps of model coefficients where the local *t*-test exceed ±1.96 (i.e., statistically significant) at each quantile of τ = 0.25, 0.50, 0.75 and 0.90 for each predictor variable (Figure 6, Figure 7 and Figure 8).

Figure 6 shows the significant model coefficients of the Building Year. Starting on τ = 0.50, it seems that the significant negative model coefficients were associated with the old residential houses. The larger the quantile was, the more negative the model coefficients were, which were more concentrated on the older houses. For example, for τ = 0.90, the larger negative model coefficients were significant on the northern side of the inner city of Syracuse where the houses built before 1920 were located (Figure 3a). Thus, those areas may have higher risk of children lead poisoning (recall that the observed 90th quantile was 0.40, indicating that 10% of these 1393 census blocks had 40% or more children’s BLL ≥ 5 µg/dL among all tested children in the census block).

Figure 7 shows the significant model coefficients of the Taxable Value, which was more important than the Building Year (Table 3). It appeared that the significant negative model coefficients of the Taxable Value were strongly related to the residential houses on the southern side of the inner city of Syracuse (Figure 3b). This local model coefficient was statistically significant across the almost entire inner city area for each quantile. Again, the larger the quantile was, the more negative the model coefficients were.

Figure 8 is the geographic distribution of significant coefficients for the soil lead concentration. Generally, the soil lead was positively related to the incident rate of children’s lead poisoning (Table 3). The observed high soil lead concentration was related to older houses with lower taxable values (Figure 3c). It seems that, for τ = 0.75, the larger positive model coefficients were significant on both northern and southern sides of the inner city of Syracuse, while for τ = 0.90, the significant positive model coefficients were more on the southern side of the inner city of Syracuse.

## 5. Discussion

Quantile regression (QR) is aimed at estimating the conditional quantiles (e.g., 5th or 90th quantiles) of response variable given the predictor variables. Compared to the conventional OLS regression which models the average relationships, QR computes several regression curves corresponding to different quantiles or percentiles of the response variable. Therefore, the feature of QR is to offer more complete ranges of statistical models than the classical conditional mean regression [38]. The regression relationships at the “average” level and at different quantiles are quite different. Shao et al. (2017) concluded that more recently built houses with higher average town taxable values would have lower likelihood of lead poisoning, while the children would have a higher chance of exposure to lead if the surrounding of the house had higher soil lead concentration for the “average” relationship via Poisson models [11]. In this study, the global quantile models confirmed that influential directions extending to both lower and upper tails. This importance pattern of the three predictor variables was different from those of the four quantile models (Table 3). In practice, when the upper or lower quantiles of the response variable are more interested to researchers or policy makers, QR is particularly useful for exploring the full range of the variable relationships [36,37]. However, the global QR only offers a picture of global regularity. The issues of local spatial constituents, structure, variability, and complexity are still undetected.

The GWQR modeling method developed by [44], which integrates QR into GWR, accounts for both spatial effects and quantile distribution of the response variable. Specifically, GWQR allows researchers to explore non-stationarity across the study area depending on various quantiles of the response variable. Those two dimensions are particularly important in the fields of epidemiology and public health. We present here a case study of environmental epidemiology, the children’s lead poisoning in Syracuse. It is well known that lead poisoning in the inner cities was caused by lead-based paint, lead in drinking water system, and lead in dust and soil. The census blocks of the inner cities were built at different time periods, so were the water pipeline systems, causing the non-stationary lead hazard exposure and clustered lead poisoning. Therefore, using localized information to study the children lead poisoning would be more precise than using all the data in the study area. Thus, we utilized GWQR to better identify the specific “high risk” spots of children lead poisoning in these at-risk neighborhoods.

The first advantage of GWQR is flexible for modeling data with heterogeneous distributions. Methodologically, it is extended from quantile regression, which makes no distributional assumption about the error term of the model. In other words, GWQR is not stick to the issues which may violate the assumptions of an OLS model such as normality. For example, in this study, about 50 percentages of blocks do not have children’s BLL ≥ 5 µg/dL. Thus, the overdispersion of the response variable violates the assumption of an OLS model, including both global and geographical weighted regression. Therefore, GWQR is unbiased when the tails and central location of the conditional distribution vary differently in the response variable [44].

Although an increasing interest in modeling the locally varying relationships has emerged in recent years, most geographically weight models are limited to link the conditional mean of the response variable to the predictor variables. However, our results indicated that the magnitudes of the estimated model coefficients of all predictor variables were very different between the 50th quantile model against the 90th quantile model (Table 5). It is noticeable that the not only the magnitudes of the model coefficients were dissimilar, but also the locations of important predictors were divergent in terms of significance tests (Figure 6, Figure 7 and Figure 8), indicating that the impacts of the environmental factors were spatially varying for different risk levels (i.e., different quantiles) of children lead poisoning. In this study, the observed 75th quantile of response variable was considered “high risk” areas of children’s lead poisoning and should be identified for lead control treatment. The important factors of soil lead consideration (Figure 8c) were located at the high-risk clusters in central blocks of Syracuse (Figure 1). This indicated that the soil concentration is strongly associated with high risks of lead poisoning. But in the previous study using a spatial hurdle model [52], the soil concentration was not strongly associated with high risks of lead poisoning via average level. Therefore, GWQR is able to help researchers to explore the locally detailed relationships simultaneously across different conditional distributions of the response variable and provide deeper insights in terms of the disease prevention and protection. The identification of those “high risk” spots would assist the government agencies to make better decisions on where and what the lead hazard treatment should focus on.

Although GWQR is an improved regression method toward a spatial quantile-based analysis, some issues still need to be further developed and discussion. The first issue is the bandwidth selection. In this study, the variogram model was fitted through the variogram values in order to find the optimal bandwidth and kernel function [53]. We tried three ways for the variograms, including the observed responsible variable, the residuals of the global OLS regression, and the residuals based on 0.25, 0.50, 0.75 and 0.90 global quantile models. All optimal models were resulted to Gaussian due to the smallest AIC. Estimated bandwidths were 2324, 2712, 2548, 2461, 2640, and 2544 m, respectively. Given the estimation of the bandwidths were very close, we chose 2324 m as the bandwidth referenced on the observed incident rate of the children’s blood lead level for the GWQR analysis. However, optimal bandwidth may be selected in terms of other criterions, such as subjective and smallest cross-validation error [54]. The better bandwidth selection method for quantile regression, which uses linear programming distinct from OLS, demands further study.

Another issue in this study was the boundary effect of spatial data analysis. In the middle of our study region, the residential census blocks were separated by the non-residential blocks (commercial centers) in the center of the Syracuse and at the edge of map (Figure 2). Therefore, some census blocks may be surrounded by the blocks defined by the bandwidth where there were no data of children’s BLL available. This could be a potential problem for fitting the GWQR models [53]. Thus, additional information from other neighborhood cities may be required to correct that edge effect and improve model explanatory range.

## 6. Conclusions

Children lead poisoning has been a concern of public health in the USA, especially in some inner cities where the residential houses are much older with poor conditions. Thus, young children may have higher risks to exposure to lead hazard. Over the last two decades researchers have been using regression models, including both global and GWR models, to investigate the relationships between children lead poisoning and environment factors. However, most regression models focus on the “average” variable relationships and provide the prediction on the conditional mean (i.e., central behavior) of the response variable. However, for the studies of environmental epidemiology, the conditional percentiles (or quantiles) of children lead poisoning may be more interesting to researchers and public agencies, because different quantiles represent different levels of “risks” of children’s exposure to lead poisoning. In this study we applied global quantile regression (QR) and geographical weighted quantile regression (GWQR) to model the relationships between children’s lead poisoning and three environmental factors at different quantiles (25th, 50th, 75th and 90th). The response variable was the incident rate of children’s BLL ≥ 5 µg/dL in each census block, and the three predictor variables were (1) building year, (2) natural logarithm of town taxable values (in thousand dollars), and (3) soil lead concentration (ppm).

Our results indicated that at each quantile, the regression coefficients of both global QR and GWQR models were (1) negative for both building year and town taxable values, indicating that the incident rate of children lead poisoning reduced with newer buildings and/or higher taxable values of the houses; and (2) positive for the soil lead concentration, implying that higher soil lead concentration around the house may cause higher risks of children’s lead poisoning. Further, these negative or positive relationships between response variable and three environmental factors became stronger for larger quantiles. The GWQR models enabled us to explore the full distribution of children’s lead poisoning and identify “high risk” locations or areas in the neighborhood of the inner city of Syracuse, NY, USA which would provide useful information to assist the government agencies to make better decisions on where and what the lead hazard treatment should focus on.

## Figures and Tables

**Table 1:** Descriptive statistics of variables used in this study (n = 1393).

Variable	Mean	Std Dev	Minimum	Maximum
Rate of BLL ≥ 5 µg/dL	0.1375	0.2075	0.0	1.0
Building Year	1923	17.6	1860	1978
Town Taxable Values (K$)	58.445	23.815	14.000	230.106
Soil Lead (ppm)	185.8	112.5	10.03	840.8

**Table 2:** Distribution summary of the response variable (the incident rate of children’s BLL ≥ 5 µg/dL).

τ	0.25	0.50	0.60	0.70	0.75	0.80	0.90	0.95	0.99	1.0
ξ(τ)	0.0	0.05	0.08	0.14	0.18	0.25	0.40	0.57	1.0	1.0

**Table 3:** Coefficient estimates of global quantile regression models for τ = 25, 50, 75 and 90.

Coefficient	Estimate	95% Confidence Limits		*p*-Value	STB ^†^
τ = 0.25					
Intercept	0.5707	−0.0234	1.1647	0.0597	
Building Year	−0.0002	−0.0005	0.0001	0.1119	−0.0201
Log Tax Values	−0.0288	−0.0393	−0.0183	<0.0001	−0.0502
Soil Lead	0.0001	0.0001	0.0002	<0.0001	0.0688
τ = 0.50					
Intercept	2.4739	1.3337	3.6141	<0.001	
Building Year	−0.0011	−0.0017	−0.0005	0.0002	−0.0914
Log Tax Values	−0.0905	−0.1120	−0.0689	<0.001	−0.1576
Soil Lead	0.0003	0.0002	0.0004	<0.001	0.1411
τ = 0.75					
Intercept	4.9176	3.6101	6.2251	<0.0001	
Building Year	−0.0021	−0.0028	−0.0015	<0.0001	−0.1813
Log Tax Values	−0.1734	−0.1954	−0.1515	<0.0001	−0.3012
Soil Lead	0.0005	0.0004	0.0006	<0.0001	0.2629
τ = 0.90					
Intercept	7.4102	4.5054	10.3150	<0.0001	
Building Year	−0.0033	−0.0047	−0.0018	<0.0001	−0.2764
Log Tax Values	−0.2303	−0.2761	−0.1844	<0.0001	−0.4011
Soil Lead	0.0007	0.0003	0.0011	0.0007	0.3715

^†^ Note: STB—standardized model coefficients.

**Table 4:** Moran’s I for the observed incident rate of children’s BLL ≥ 5 µg/dL and the residuals of global quantile regression models.

Test	Observed (BLL ≥ 5 µg/dL)	Residuals τ = 0.25	Residuals τ = 0.50	Residuals τ = 0.75	Residuals τ = 0.90
**Moran’s Index**	0.0974	0.0782	0.045	0.02	0.0276
**Z-score**	69.10	55.56	32.20	14.61	19.91
***p*** **-value**	<0.0001	<0.0001	<0.0001	<0.0001	<0.0001

**Table 5:** Summary of parameter estimates of GWQR models for τ = 25, 50, 75 and 90.

Coefficient	Mean	Median	Min	Max	IQR	Ste ^†^	Status
τ = 0.25							
Intercept	0.5409	0.5996	−0.8802	1.3253	0.7439	0.3028	Nonstationary
Building Year	−0.00021	−0.0002	−0.0007	0.0006	0.0004	0.0001	Nonstationary
Log Tax Values	−0.0315	−0.0279	−0.0879	0.0398	0.0453	0.0055	Nonstationary
Soil Lead	0.000095	0.0001	−0.0001	0.0003	0.0000	0.0000	Stationary
τ = 0.50							
Intercept	2.3455	2.4911	−0.9819	5.0047	1.7971	0.5812	Nonstationary
Building Year	−0.001051	−0.0011	−0.0024	0.0006	0.0008	0.0003	Nonstationary
Log Tax Values	−0.07374	−0.0615	−0.2094	0.0657	0.0959	0.0110	Nonstationary
Soil Lead	0.000176	0.0002	−0.0001	0.0005	0.0001	0.0000	Nonstationary
τ = 0.75							
Intercept	3.7948	4.1557	−4.4179	8.8531	3.5624	0.6665	Nonstationary
Building Year	−0.001703	−0.0018	−0.0043	0.0022	0.0002	0.0003	Nonstationary
Log Tax Values	−0.1102	−0.0975	−0.2993	0.0994	0.1244	0.0112	Nonstationary
Soil Lead	0.000299	0.0003	−0.0003	0.0006	0.002	0.0001	Nonstationary
τ = 0.90							
Intercept	4.0228	3.6329	−10.0867	12.0888	6.2613	1.4808	Nonstationary
Building Year	−0.001726	−0.0014	−0.0059	0.03699	0.0033	0.0008	Nonstationary
Log Tax Values	−0.1406	−0.1413	−0.3871	0.1247	0.1589	0.0204	Nonstationary
Soil Lead	0.000444	0.0004	−0.0006	0.1342	0.0003	0.0002	Stationary

^†^ Note: Standard error (Ste) was estimated from the global quantile regression.
